# Risk Factors and Prevention Strategies for Postoperative Opioid Abuse

**DOI:** 10.1155/2019/7490801

**Published:** 2019-07-10

**Authors:** Shuai Zhao, Fan Chen, Anqi Feng, Wei Han, Yuan Zhang

**Affiliations:** ^1^Department of Anesthesiology, First Hospital of Jilin University, Changchun, China; ^2^Department of Neurosurgery, First Hospital of Jilin University, Changchun, China; ^3^Department of Anesthesiology, Second Hospital of Xi'an Jiaotong University, Xi'an, China

## Abstract

Worldwide, 80% of patients who undergo surgery receive opioid analgesics as the fundamental agent for pain relief. However, the irrational use of opioids leads to excessive drug dependence and drug abuse, resulting in an increased mortality rate and huge economic loss. The crisis of opioid overuse remains a great challenge. In this review, we summarize several key factors in opioid abuse, including race, region, income, genetic factors, age and gender, smoking and alcohol abuse, history of chronic pain and analgesic drug abuse, surgery, neuropsychiatric illness, depression and antidepressant use, human factors, national policies, hospital regulations, and health insurance under treatment of pain. Furthermore, we present several prevention strategies, such as perioperative measures, opioid substitutes, treatment of the primary illness, emotional regulation, use of opioid antagonists, efforts of the state, hospitals, doctors and pharmacy benefit managers, gene therapy, and vaccines. Greater understanding and better assessment are required of the risks associated with opioid abuse to ensure the safety and analgesic effects of pain treatment after surgery.

## 1. Introduction

**Figure 1:**
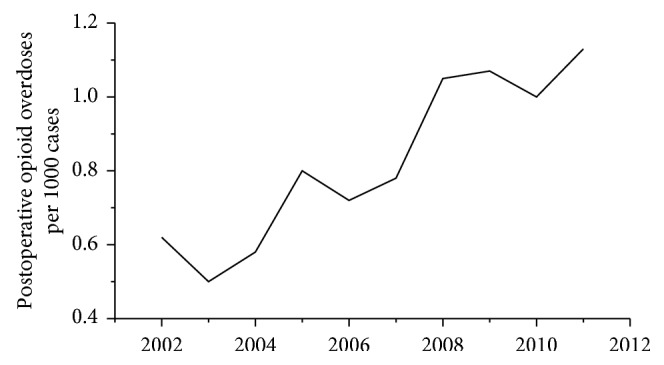
Postoperative opioid overdoses by year. The proportion of patients with postoperative opioid overdoses increased from 0.6/1000 in 2002 to 1.1/1000 in 2012. This figure was adapted from the study by Cauley et al. [24].

Despite the increasing use of minimally invasive surgeries [1–3] and new medications, opioid analgesics are still considered to be among the basic agents for treatment of pain after surgery. Eighty percent of patients who undergo surgery receive opioids for postoperative pain relief [4]. Many studies have confirmed that satisfactory analgesia promotes recovery and improves postoperative outcomes [5–8]. Unfortunately, opioids can also lead to many adverse reactions. Over the previous decades, opioid-related increased risks for opioid misuse, abuse, dependence, addiction, and overdose deaths have become a great concern. These serious events result in increased postoperative morbidity and mortality and healthcare resource utilization. This dangerous trend is rapidly increasing by year [9–24], as shown in Figure 1.

In the United States, deaths related to opioid exposure have reached 33,000 per year [25–27]. This exceeds the number of deaths from both motor vehicle accidents and acquired immune deficiency syndrome (AIDS) [28, 29]. Notably, opioid-related hospitalizations have shifted from diagnoses of opioid abuse or dependence to those with opioid intoxication [22]. Moreover, substantial financial expenditure is needed for the treatment of drug addiction and drug overdoses in the United States: the costs of opioid use, abuse, and overdose amounted to $78.5 billion in 2013 [28]. Therefore, it is a great challenge to control and prevent the serious consequences of the current opioid crisis.

A larger number of studies have reported the risk factors for opioid abuse [24, 30, 31]. However, up to now, no systematic and comprehensive review has focused on the prevention strategies that are currently used to reduce opioid misuse and abuse, with limited significant guidance for clinical practice. Prior to pain therapy using opioids, a clinician should always assess the risks for opioid use disorders in order to establish the appropriate monitoring strategy and the optimal usage [30]. In this review, we summarize the risk factors for opioid abuse and the current prevention measures, based on the latest evidence. We highlight the importance for the clinicians to provide a safe and effective pain relief treatment that is tailored to each patient, aiming to minimize opioid-related risks, improve patient outcomes, and reduce healthcare resource utilization.

## 2. Risk Factors for Opioid Abuse

### 2.1. Race, Region, and Income

According to a recent study, African Americans experience more acute pain than non-Hispanic white patients in a variety of acute medical situations. Furthermore, African Americans are more likely to receive nonsteroidal anti-inflammatory drugs (NSAIDs) instead of opioids. However, this racial difference, in the severity of acute posttraumatic pain, may not be explained by factors of socioeconomic status or accident characteristics [32]. Another study by Swenson et al. [33] included 24,331 women and showed that African American race was an independent risk factor for new persistent opioid use following hysterectomy. Based on National Medicare data from 2007 to 2012, Kuo et al. [34] assessed the risk factors of opioid use and found that opioid use was associated with race. Regarding region and income, Cauley et al. [24] suggested that these two factors are also related to opioid overuse. Several studies have confirmed that a low socioeconomic position contributes to opioid abuse [21, 35]. Clarke et al. [35] studied 39,140 patients and found that low-income patients required more opioids in the 90-day period following surgery.

In China, there is no solid evidence to show the effects of race, region, and income factors on opioid abuse. However, clinical experience indicates a relatively high incidence of opioid abuse in minority ethnic groups with low incomes, those who live in remote areas, and, especially, those who earn their living through manual labor.

### 2.2. Genetic Variability

Several studies have identified a number of genes that are related to opioid use. Smith et al. [36] found a single genome-wide significant association with methadone dose and the closest gene *OPRM1*. In another study, De Gregori et al. [37] demonstrated that a combination of genetic allelic variants within the *OPRM1*, *COMT*, and *ESR1* genes substantially influenced morphine consumption after abdominal surgery. This highlights the important contribution of genetic variability in postoperative analgesia.

Kringel et al. summarized the effects of gene polymorphism on opioid use and pain, as displayed in Table 1 [46]. In addition, Donaldson et al. [47] found that the multiple PDZ domain protein Mpdz/MUPP1 may regulate opioid tolerance and opioid-induced hyperalgesia, possibly through an indirect action. However, a recent study demonstrated no major relationship between single nucleotide polymorphisms (SNPs) of OPRM1, ABCB1, COMT and morphine consumption, pain severity, or adverse events in the postoperative period in white patients undergoing major orthopedic surgery. In this study, only P-glycoprotein polymorphisms (ex-21; ex-26) were significantly related to morphine concentration in the postanesthesia care unit (PACU). However, the very low coefficient suggests the poor prediction capability of this model [48].

Taken together, some studies have reported a correlation between certain genes and opioid consumption, while others have shown different results. Therefore, further studies are needed to investigate the relationship between genetic viability and opioid use.

### 2.3. Age and Gender

Previous studies have indicated that elderly patients are more likely to develop persistent opioid use after surgery [34]. One study showed that an age >80 years was a risk factor for excessive opioid use [21]. Another study identified the point at which age became a risk factor for excessive opioid use was >50 years [49]. Swenson et al. analyzed the risk factors for new persistent opioid use after hysterectomy and found that increasing age was an independent risk factor [33]. However, a younger age has also been shown to be associated with postoperative opioid overuse. Clarke et al. followed 39,140 patients for 90 days after surgery and found that opioid use was higher in the younger age group [35]. Furthermore, gender is also a contributing factor associated with opioid overuse [21]. Some studies have indicated that women are at a higher risk for opioid use than men [21]. However, chronic opioid use has also been reported in male patients during the postoperative period [49]. The results from previous studies vary in terms of the impact of age and gender on opioid use. Thus, more research on this topic is required.

### 2.4. Smoking and Alcohol Abuse

It has been reported that nicotine dependence is associated with increased sensitivity to pain [50–52]. Data from clinical observations demonstrate that patients with nicotine dependence or that are living in a second-hand smoking environment consumed a larger quantity of opioids after surgery, or in their daily life, than nonsmokers [53, 54]. Smoking is considered to be an important and modifiable factor in opioid use [55]. Moreover, many studies have shown that long-term tobacco dependence leads to a higher incidence of postoperative chronic pain in the back, skeletal muscles, joints, and peripheral nerves [56–65]. Regarding alcohol abuse, data from several studies demonstrate that alcoholics have a high risk of opioid overuse after surgery [29, 49, 54].

### 2.5. History of Chronic Pain and Analgesic Drug Abuse

Chronic pain is a major contributing factor for increased opioid consumption. In Australia, patients with osteoarthritis are the main consumers of opioids, which results in a huge medical burden [66]. In addition to osteoarthritis, rheumatoid arthritis is another risk factor for opioid overuse [21, 32, 33]. For patients with a history of daily opioid abuse, a higher dose of opioids is needed after surgery [24]. Olfson et al. [67] conducted a 3-year follow-up study to determine whether cannabis use was associated with nonmedical opioid use and opioid use disorders. Contrary to the preliminary hypothesis, they found that cannabis use appeared to increase rather than decrease the risk of developing nonmedical prescription of opioids and opioid use disorders. In addition, Brummett et al. [54] suggested that novel persistent opioid use after surgery is common, with no significant difference between opioid use following minor and major surgical procedures. However, they also found that this persistent opioid use was associated with behavioral and pain disorders. In patients undergoing cesarean section, Fox et al. [20] reported that preoperative opioid abuse was a predictive factor for the continued use of opioids after surgery.

### 2.6. Surgery

**Figure 2:**
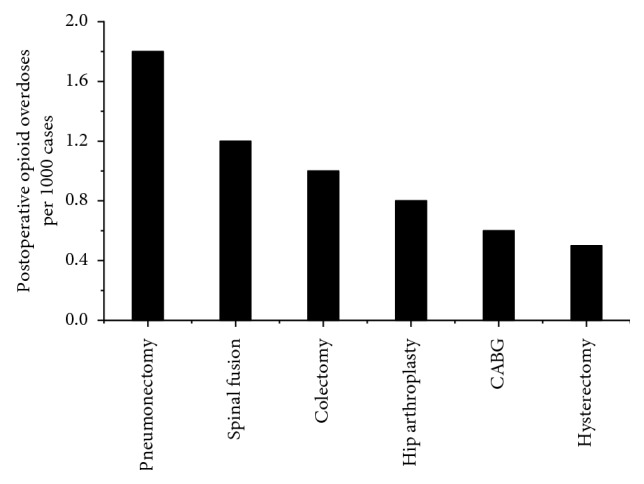
Postoperative opioid overdoses by surgery type. The proportion of patients with postoperative opioid overdoses following different surgical procedures. The highest risk occurred following pneumonectomy (1.8/1000), followed by spinal fusion (1.2/1000). This figure was adapted from the study by Cauley et al. [24].

According to a study on the global volume of surgery in 2012, more than 300 million patients undergo surgical procedures each year [68]. Pain is a leading cause of opioid use disorder and opioid prescription after surgery [69, 70]. The incidence of opioid abuse varies following different types of surgeries. In a large-scale retrospective analysis, Sun et al. [49] included 641,941 opioid-naive surgical patients and 18,011,137 opioid-naive nonsurgical patients. Their results demonstrate that except for cataract surgery, laparoscopic appendectomy, FESS, and TURP, many surgical procedures are associated with an increased risk of chronic opioid use during the postoperative period.  Table 2 displays data regarding postoperative opioid overdoses following different types of surgery. According to the study by Cauley et al. [24], the highest risk of opioid overuse occurred in pneumonectomy (1.8/1000), followed by spinal fusion (1.2/1000), as shown in Figure 2. In addition, specific comorbidities can also increase the risk of opioid overuse after surgery. Clarke et al. [35] studied 39,140 patients and found that patients with preexisting conditions, including diabetes, heart failure, and lung disease, received a higher dose of opioids during the 90-day postoperative period.

**Figure 3:**
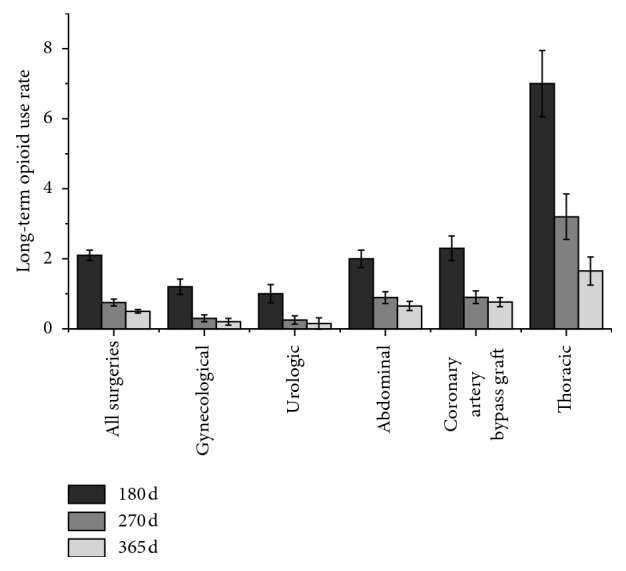
Long-term opioid use after major elective surgery. The proportion of patients with long-term opioid use at postoperative days 180, 270, and 365 following different surgical procedures. The highest risk occurred following thoracic surgery (1.7% at day 365). This figure was adapted from the study by Soneji et al. [77].

**Figure 4:**
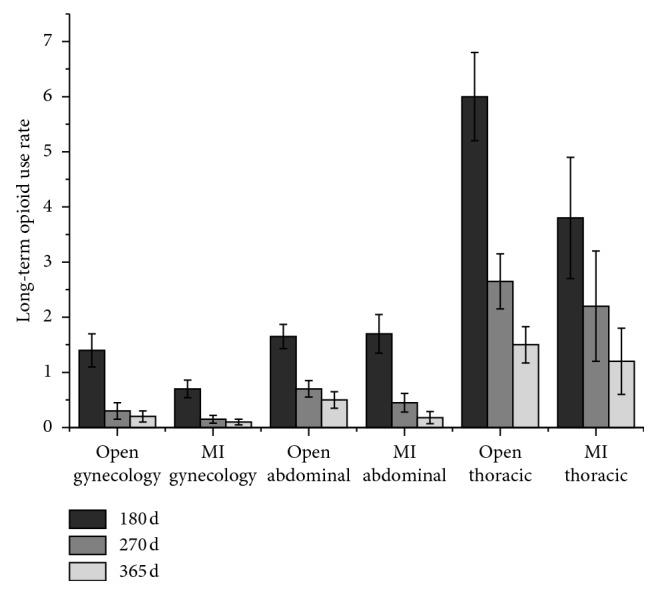
Long-term opioid use after open surgery vs. MI surgery. The proportion of patients with long-term opioid use at postoperative days 180, 270, and 365 after open surgery vs. MI surgery. The highest risk occurred following thoracic surgery (at day 365, 1.7% in open surgery and 1.3% in MI surgery). MI = minimally invasive. This figure was adapted from the study by Soneji et al. [77].

Soneji et al. [77] reviewed the data of 39,140 Canadian elderly patients and investigated the outcomes related to opioid use during a 365-day postoperative follow-up period. As shown in Figure 3, the results suggest that the proportion of patients with long-term opioid use at postoperative days 180, 270, and 365 varied between the different surgical procedures. The highest risk of long-term opioid use occurred following thoracic surgery (1.7% at postoperative day 365). This study also found that patients undergoing open surgery required more opioids after surgery, with the highest risk observed following thoracic surgery (1.7% following open surgery and 1.3% following MI surgery at postoperative day 365), as shown in Figure 4 [77]. In addition, Lee et al. analyzed 1-year of follow-up data from 68,463 patients that underwent various surgeries. They noted that patients who received chemotherapy had an increased risk of new persistent opioid use [73].

### 2.7. Neuropsychiatric Disorders, Depression, and Use of Antidepressants

Previous studies have shown that a history of psychiatric disorders and benzodiazepine use is associated with opioid overuse [20]. During a 90-day follow-up period after surgery in 39,140 patients, Clarke et al. [35] found that patients with preoperative benzodiazepine use required more postoperative opioids than patients with no preoperative benzodiazepine use.

Sun et al. [49] conducted a clinical observation indicating that use of benzodiazepines or antidepressants was associated with a long-term opioid use in surgical patients. In a retrospective analysis that included 315,428 privately insured patients in the US, concurrent benzodiazepine/opioid use markedly increased from 2001 to 2013. This increase significantly contributed to the overall population risk of opioid overdose [92]. Another study by Quinn et al. [93] found evidence that risk of greater long-term opioid receipt was associated with psychiatric and behavioral conditions among commercially insured patients. Consistent with these findings, other studies have reported that patients with Alzheimer disease (AD), depression, mood disorders, and polyneuropathy can develop long-term opioid use in their daily life [21, 94].

### 2.8. Human Factors

Finkelstein's study [95] found that children of mothers who are prescribed opioids are at a markedly increased risk of opioid abuse. Kumar et al. [96] found that most outpatient shoulder surgery patients were prescribed more opioid analgesics than they consumed and there was a lack of patient education regarding the disposal of opioids.

Conversely, Maughan et al. [97] demonstrated a reduction in the opioid use within 21 days after a tooth extraction through providing patients with opioid use instructions. In addition, pharmaceutical companies may bribe doctors to prescribe more opioids, which is also a factor of opioid overuse. In one investigation, the use of mucosal fentanyl that was related to bribery accounted for 40% of all of the prescriptions in the US. Unfortunately, according to the Food and Drug Administration reports, at least 63 patients have died from an overdose or other complications associated with Subsys [98].

### 2.9. National Policies and Healthcare Insurance

Medical insurance and subsidies may be involved in opioids overuse. It has been shown that patients with medical assistance [34, 99] consume more opioids than those without medical assistance after a tooth extraction [100]. National Medicare data from 2007–2012 demonstrate that state law regulating pain clinics were associated with a reduction in schedule II opioid prescriptions [34]. In addition, prescription drug monitoring programs (PDMPs) may contribute to the observed reductions in opioid overuse [101]. However, opposing opinions exist [102]. Meara et al. investigated the association between prescription-opioid receipt and state-controlled laws by analyzing data from 8.7 million disabled people between 2006 and 2012. This study concluded that the adoption of controlled-substance laws was not associated with a reduction in the potentially hazardous use of opioids or overdose among disabled Medicare beneficiaries. Despite various investigations in this area, the majority of studies support a role of national medical policies and insurance in opioid use. More relevant studies are needed to explore the risk factors of opioid overuse in order to make effective strategies for prevention.

## 3. Prevention Strategies for Opioid Abuse

The reduction of opioid overuse is an urgent task for clinicians worldwide. Some people are worried that restricted opioid dosing guidelines might compromise the effect of opioid therapy for patients who could benefit from it. However, the risks associated with high-dose opioid therapy outweigh the benefits [74]. Therefore, more studies are needed to investigate the optimal methods for opioid misuse reduction.

### 3.1. Perioperative Interventions

Worldwide, more than 300 million patients undergo surgical treatment each year [68]. Pain is considered to be a predictor of opioid use disorder (OUD) and opioid prescription [69, 70]. Notably, the postoperative period is a specific time when patients are susceptible to OUD. New persistent opioid use represents a common but previously underreported condition related to surgical intervention that warrants more awareness [35, 103, 104]. Therefore, management of the occurrence and development of postoperative pain is a key step to prevent opioid abuse.

#### 3.1.1. Enhanced Recovery after Surgery (ERAS)

ERAS is a promising way to reduce opioid use after surgery [71]. A recent study by Meyer et al. studied more than 600 patients following gynecological surgery. They found that the ERAS group had a 72% reduction in median opioid consumption and 16% of patients receiving ERAS were opioid-free from hospital admission up to the third postoperative day [105]. Therefore, it has been suggested that ERAS strategies should be utilized to reduce opioid abuse as well as promote the rehabilitation of patients.

#### 3.1.2. Regional Block, Local Infiltration, and Acupuncture

Regional block of pain and infiltration with local anesthetics play an important role in pain control as well as in reducing the risk of opioid abuse after surgery. The benefits of these strategies have been demonstrated by a large number of studies following various surgical procedures, including total knee arthroplasty (TKA), ligamentum arthrodesis, hepatectomy, lumpectomy, colposcopy, and endoscopic surgeries [85].

In addition, the combination of intravenous sedatives and acupuncture procedures can produce satisfactory postoperative analgesia, which may help to reduce the risk of opioid overuse. In a Cochrane review, Kwan et al. [106] included 24 randomized controlled trials (RCTs; from 3160 participants) to evaluate several combinations of sedation and analgesia for pain relief and pregnancy outcomes in women who underwent oocyte retrieval. This study found that the simultaneous use of sedation, opioid analgesia, and nerve block or acupuncture techniques resulted in better pain relief compared to any single intervention.

However, Ladha et al. [107] analyzed the use of opioids within 90 days of discharge in 6,432 patients undergoing abdominal surgery and found that epidural analgesia did not significantly reduce persistent opioid use in the postoperative period. This discrepancy calls for more studies to investigate the mechanism of postoperative persistent opioid use as well as its prevention.

#### 3.1.3. Multimode Intravenous Analgesia

Perioperative multimodal analgesia plays an important role in the prevention of opioid abuse after surgery. The combination of nonsteroidal anti-inflammatory (NSAID), dexmedetomidine, and weak opioids can produce satisfactory analgesia without significant adverse effects related to opioids [81, 82, 87–89]. However, some medications should be used with caution. In patients undergoing cesarean section, the administration of gabapentin did not improve postoperative analgesia but led to a higher incidence of sedation [108]. The use of gabapentin and pregabalin in multimodal analgesia is still controversial [109, 110].

Many surgeons and anesthesiologists prefer to use NSAIDs to control mild pain after surgery. While NSAIDs are beneficial to many patients, safety concerns are increasing as their use becomes more prevalent. Related negative effects, such as aggravating digestive ulcer and heart and kidney effects are needed to be considered, especially for elderly patients. To optimize safety and efficacy, its use should always be tailored to each individual patient. In addition, all patients prescribed NSAIDs should be monitored for gastrointestinal, cardiovascular, and renal functions [111]. When applying multimodal analgesia, it is very important to keep the related side effects in mind in order to prioritize the safety of the patient.

### 3.2. Perioperative Physicians

It is the job of doctors to monitor opioid abuse. However, doctors can also be opioid abusers [14, 29]. The role of surgical prescription in the opioid epidemic has gained increasing recognition. Vu and Lin [29] demonstrated that surgeons may contribute to the control of opioid misuse in three stages: preoperatively, intraoperatively, and postoperatively.

Sensory dysfunction is a common symptom of neuropathic pain. Nerve injury as a result of surgical manipulation is a leading cause of neuropathic pain after surgery [112]. Swenson et al. found that abdominal surgery was associated with new and sustained opioid use after hysterectomy, suggesting a reasonable route of surgery should be taken into consideration for reducing the risk of opioid overuse [33]. Therefore, surgeons are expected to prioritize the protection of nerves during meticulous operations.

A large number of surgical patients are nicotine dependent. Chiang et al. [72] demonstrated that patients who quit smoking before surgery needed a lower dose of postoperative opioids than those who did not stop smoking. This indicates the effect of preoperative smoking cessation on postoperative opioid consumption in patients with nicotine dependence. Therefore, patients who are current smokers are encouraged to stop smoking before surgery.

In addition, other medical workers, such as pharmacy benefit managers (PBM) help to limit opioid medication in opioid-naive patients to 7 total days for initial prescription, improving safety and convenience for consumers and employers as well as reducing prescription drug costs. Thus, efforts from PBM also play a positive role in the control of opioid abuse [113].

In summary, doctors play an important part in perioperative opioid use. More efforts are needed to control the occurrence and development of pain during the preoperative, intraoperative, and postoperative periods.

### 3.3. Alternative Agents to Opioids

Gabapentin/pregabalin, another first-line drug for the treatment of neuropathic pain has been used for postoperative pain control [108, 114]. A randomized, double-blind clinical study found that perioperative administration of gabapentin had no effect on postoperative pain resolution, but it had a modest effect on promoting opioid cessation after surgery. Thus, the use of perioperative gabapentin may help to promote opioid cessation and prevent chronic opioid use [115]. However, it should be noted that the combination of gabapentin and opioids appeared to result in an increased risk of mortality [114]. Therefore, the safety and efficacy of these opioid substitutes have not been fully tested in the clinical setting and further investigations on this topic are required.

### 3.4. Opioid Agonist

Opioid substitutes are agents which are expected to produce equivalent analgesic effects to opioids without opioid-related side effects or the risk of opioid abuse.

A recent study demonstrated the efficacy of kappa opioid receptor (KOR) agonists in the treatment of cancer-induced bone pain in mice, without changing the tumor size or affecting cancer cell proliferation. This suggests that KOR agonists may be a promising target for cancer pain management [116].

Methadone and buprenorphine are currently used to treat opioid use disorders. These drugs also appear to help reduce suicidality and crime [117]. Recently, Haumann et al. reported that methadone was superior to fentanyl in the treatment of neuropathic pain in patients with head-and-neck cancer. Nicholson et al. conducted a comprehensive review to demonstrate the role of methadone in cancer pain treatment. Therefore, for some types of cancer pain and neuropathic pain, opioid agonist may be used with satisfactory therapeutic effects to minimize the risk of opioid overuse [82, 118]. Results from animal experiments should also be considered regarding the effects of opioid use on the survival of cancer patients. Boland et al. indicated that buprenorphine does not affect NK cell autonomy. However, tramadol has been found to increase NK cell cytotoxicity and reduce tumor metastasis [119].

More studies are still needed to clarify the effects and mechanisms of these nonopioid analgesics on cancer pain. In addition, it should be noted that death caused by methadone overdose has shown a tendency to increase in the United States [120].

### 3.5. Primary Disease Control

Control of the primary disease may help to reduce opioid consumption after surgery. Previous studies have suggested that opioids should not be used as the first-line treatment for peripheral neuropathic pain or polyneuropathy, resulting in a limited therapeutic effect and increased risk of drug dependence and overdose [121, 122]. In patients with mental disorders, disease development is often associated with excessive opioid use [123]. Hooten et al. [76] conducted a review and reported that chronic pain and mental health disorders are common in the general population. In addition, this review suggested that a bidirectional relationship exists between these two pathophysiologic conditions. Despite the fact that pharmacological management of concomitant pain and mental health disorders is often challenging, several other agents, including serotonin-norepinephrine reuptake inhibitors, tricyclic antidepressants, and anticonvulsants may be considered to be the first-line drugs for treatment in these cases. Thus, for patients with opioid abuse caused by various diseases, the first step of treatment is to treat the primary disease itself rather than to simply deal with the pain; otherwise, the risk of drug abuse will arise.

### 3.6. Mood Control

The experience of pain often leads to the occurrence and development of negative emotions. Conversely, the control of emotions, such as a state of relaxation, may help to relieve pain and prevent drug abuse. In a randomized controlled trial that studied pain management during childbirth, Smith et al. [91] reported that relaxation, yoga, and music therapy may play a role in pain reduction patient satisfaction. In addition, acceptance and commitment therapy (ACT) reduced struggle against difficult inner experiences (such as pain), fostered long-term behavior patterns, and reduced the risk of opioid misuse [124, 125]. Therefore, for patients with chronic pain and negative mood, mental care and encouraging words may not only help them relax but may also reduce and prevent opioid abuse.

### 3.7. Opioid Antagonists

Opioid antagonists can be used to prevent opioid overuse. A recent study demonstrated that intranasal naloxone administration has the potential to be part of evolving clinical practice, in terms of opioid overdose prevention [90]. However, another study found that intranasal naloxone did not reduce opioid-related adverse events [126]. Therefore, for patients with a high risk of opioid overuse, intranasal naloxone may be helpful. However, more evidence is needed to support the use of naloxone in this context.

### 3.8. Government Policies, Hospital Regulations, and Education of Medical Workers

Government policies, hospital regulations, and education of medical workers are also important factors in the control of opioid abuse. The formulation and implementation of education and guidelines for opioid treatment, as well as the prescription and clinical guidance of medical workers, play a positive role in the prevention of opioid overuse [73, 74, 75]. Several steps can be taken to prevent opioid abuse [127]. Government managers and policy makers should consider revising the indications for opioid use in chronic pain treatment, as well as restricting or eliminating the marketing of opioids. Next, medical insurance coverage should be improved to encourage nonopioid and nonpharmacological management of pain. In addition, the supply of heroin and illicitly produced synthetic opioids should be strictly prohibited by improving coordination between legal and public health authorities.

### 3.9. New Technologies and Therapeutic Methods

Hurd et al. [128] examined the strategies that targeted specific genetic and epigenetic factors, along with novel nonopioid medications, which showed promising therapeutic potential for the prevention of opioid abuse. Another study by Olson and Janda [129] put forward the novel idea of opioid vaccines for the prevention of opioid abuse.

New technologies and therapeutic approaches, such as vaccines and gene therapy, may fundamentally alter the opioid abuse epidemic; however, there is still a long way to go in this area of study.

## 4. Summary

Worldwide, the epidemic of opioid abuse has been growing every year, resulting in disastrous consequences. There is an urgent need for governments, institutions, and each clinical practitioner to find effective means to prevent the current crisis. Therefore, a deep understanding of the prevalence and risk factors of opioid abuse is needed. Furthermore, pain issues must be dealt with a vigilant manner in such patient populations. National policies and regulations, hospital regulations, and doctors should play a critical role in this campaign for the reduction of opioid abuse. Of course, more scientific research is needed to identify more effective measures for prevention, possibly by targeting specific genes and developing vaccines, to benefit the maximal number of patients with the least possible side effects.

## Figures and Tables

**Table 1:** Variants of opioid receptor genes related to opioid-based analgesic therapy.

Gene	Clinical outcomes	Reference
OPRD1	Increased effect sizes of pain	[38]
OPRK1	Heroin addiction; alcohol dependence	[39]
SIGMAR1	Increased risk for developing Alzheimer's disease; decreased effects in response to antidepressants	[40]
OPRM1	Heroin and cocaine addiction	[41]
	Decreased effects in response to opioids	[42]
	Heroin addiction; opioid dependence	[43]
	Change-in-libido and insomnia side effects	[44]
	Increased risk in coronary heart disease	[45]
	Decreased effects in response to antidepressants	[42]

**Table 2:** Summary of perioperative prevention strategies for opioid abuse.

Preoperative	Reference
Risk factors assessment	
Substance use disorder (opioids, alcohol, tobacco and others)	[24, 50–52] [29, 49, 54]
Previous or current opioid use (>50 oral morphine equivalents)	[29]
Long-acting/extended release opioid formulations	[29]
Use of benzodiazepines and other sedatives or history of mental illness	[20]
Arthritis, depression, diabetes, heart failure, and lung disease	[21, 34, 54, 66]
Low income or living in less developed areas	[21, 35]
Prescription drug monitoring program for multiple opioid prescriptions or other agents	[29]
Suggestions	
Utilization of ERAS	[71]
Cessation of smoking	[72]
Education of patients and their families regarding the opioid-related risks, with more consultation service	[73, 74, 75]
Treatment of the primary disease, such as arthritis, depression, and mental illness	[76]
Presetting of acceptable postoperative pain level to reduce panic and tension	[29]
Detailed and well-planed surgical approach	[77]
Intraoperative	
Meticulous surgical procedures that minimize nerve damage	[78]
Advanced intraoperative monitoring	[79, 80]
Combination of several anesthesia methods and analgesic drugs	[81, 82, 83, 84]
Utilization of ERAS	[71]
Postoperative	
Implementation of educational programs and clinical guidelines on opioid use	[73, 74, 75]
Utilization of REAS	[71]
Nerve block technique or epidural blockade for postoperative analgesia	[85, 86]
Decreased use of opioids and increased use of nonopioid medications for postoperative analgesic, including NSAIDs or opioid substitutes	[81, 82, 87–89]
Use of naloxone when needed	[90]
Treatment of primary disease itself especially in patients with mental illnesses	[76]
Relaxation and minimization of anxiety	[91]

## References

[1] Bendixen M., Jørgensen O. D., Kronborg C., Andersen C., Licht P. B. (2016). Postoperative pain and quality of life after lobectomy via video-assisted thoracoscopic surgery or anterolateral thoracotomy for early stage lung cancer: a randomised controlled trial. *The Lancet Oncology*.

[2] Bayman E. O., Parekh K. R., Keech J., Larson N., Weg M. V., Brennan T. J. (2018). Preoperative patient expectations of postoperative pain are associated with moderate to severe acute pain after VATS. *Pain Medicine*.

[3] Bayman E. O., Parekh K. R., Keech J., Selte A., Brennan T. J. (2017). A prospective study of chronic pain after thoracic surgery. *Anesthesiology*.

[4] Wunsch H., Wijeysundera D. N., Passarella M. A., Neuman M. D. (2016). Opioids prescribed after low-risk surgical procedures in the United States, 2004–2012. *JAMA*.

[5] Abdallah F. W., Morgan P. J., Cil T. (2014). Ultrasound-guided multilevel paravertebral blocks and total intravenous anesthesia improve the quality of recovery after ambulatory breast tumor resection. *Anesthesiology*.

[6] Takagi K., Yoshida R., Yagi T. (2019). Effect of an enhanced recovery after surgery protocol in patients undergoing pancreaticoduodenectomy: a randomized controlled trial. *Clinical Nutrition*.

[7] Azhar R. A., Bochner B., Catto J. (2016). Enhanced recovery after urological surgery: a contemporary systematic review of outcomes, key elements, and research needs. *European Urology*.

[8] Gan T. J. (2017). Poorly controlled postoperative pain: prevalence, consequences, and prevention. *Journal of Pain Research*.

[9] Post Z. D. (2015). CORR insights®: preoperative opioid misuse is associated with increased morbidity and mortality after elective orthopaedic surgery. *Clinical Orthopaedics and Related Research*.

[10] Pathan S. A., Mitra B., Cameron P. A. (2018). A systematic review and meta-analysis comparing the efficacy of nonsteroidal anti-inflammatory drugs, opioids, and paracetamol in the treatment of acute renal colic. *European Urology*.

[11] Camilleri M., Lembo A., Katzka D. A. (2017). Opioids in gastroenterology: treating adverse effects and creating therapeutic benefits. *Clinical Gastroenterology and Hepatology*.

[12] Els C., Jackson T. D., Kunyk D. (2017). Adverse events associated with medium- and long-term use of opioids for chronic non-cancer pain: an overview of Cochrane reviews. *Cochrane Database of Systematic Reviews*.

[13] Verberkt C. A., van den Beuken-van Everdingen M. H. J., Schols J. M. G. A. (2017). Respiratory adverse effects of opioids for breathlessness: a systematic review and meta-analysis. *European Respiratory Journal*.

[14] Shafi S., Collinsworth A. W., Copeland L. A. (2018). Association of opioid-related adverse drug events with clinical and cost outcomes among surgical patients in a large integrated health care delivery system. *JAMA Surgery*.

[15] Brat G. A., Agniel D., Beam A. (2018). Postsurgical prescriptions for opioid naive patients and association with overdose and misuse: retrospective cohort study. *British Medical Journal*.

[16] Cao S., Karmouta R., Li D. G., Din R. S., Mostaghimi A. (2018). Opioid prescribing patterns and complications in the dermatology medicare population. *JAMA Dermatology*.

[17] Ishida J. H., McCulloch C. E., Steinman M. A., Grimes B. A., Johansen K. L. (2018). Opioid analgesics and adverse outcomes among hemodialysis patients. *Clinical Journal of the American Society of Nephrology*.

[18] Katakami N., Oda K., Tauchi K. (2017). Phase IIb, randomized, double-blind, placebo-controlled study of naldemedine for the treatment of opioid-induced constipation in patients with cancer. *Journal of Clinical Oncology*.

[19] Lee L. A., Caplan R. A., Stephens L. S. (2015). Postoperative opioid-induced respiratory depression. *Anesthesiology*.

[20] Fox L. M., Hoffman R. S., Vlahov D., Manini A. F. (2018). Risk factors for severe respiratory depression from prescription opioid overdose. *Addiction*.

[21] Hamina A., Taipale H., Tanskanen A. (2017). Long-term use of opioids for nonmalignant pain among community-dwelling persons with and without alzheimer disease in Finland. *Pain*.

[22] Song Z. (2017). Mortality quadrupled among opioid-driven hospitalizations, notably within lower-income and disabled white populations. *Health Affairs*.

[23] Gaither J. R., Leventhal J. M., Ryan S. A., Camenga D. R. (2016). National trends in hospitalizations for opioid poisonings among children and adolescents, 1997 to 2012. *JAMA Pediatrics*.

[24] Cauley C. E., Anderson G., Haynes A. B., Menendez M., Bateman B. T., Ladha K. (2017). Predictors of in-hospital postoperative opioid overdose after major elective operations. *Annals of Surgery*.

[25] Seth P., Scholl L., Rudd R. A., Bacon S. (2018). Overdose deaths involving opioids, cocaine, and psychostimulants—United States, 2015-2016. *MMWR. Morbidity and Mortality Weekly Report*.

[26] Rudd R. A., Aleshire N., Zibbell J. E., Gladden R. M. (2016). Increases in drug and opioid overdose deaths—United States, 2000–2014. *MMWR. Morbidity and Mortality Weekly Report*.

[27] Soelberg C. D., Brown R. E., Vivier D. D., Meyer J., Ramachandran B. (2017). The US opioid crisis. *Anesthesia & Analgesia*.

[28] Friedmann P. D., Andrews C. M., Humphreys K. (2017). How ACA repeal would worsen the opioid epidemic. *New England Journal of Medicine*.

[29] Vu J., Lin L. (2018). Opioid overdose—the surgeonʼs role. *Annals of Surgery*.

[30] Webster L. R. (2017). Risk factors for opioid-use disorder and overdose. *Anesthesia & Analgesia*.

[31] Terplan M. (2017). Women and the opioid crisis: historical context and public health solutions. *Fertility and Sterility*.

[32] Beaudoin F. L., Gutman R., Zhai W. (2018). Racial differences in presentations and predictors of acute pain after motor vehicle collision. *Pain*.

[33] Swenson C. W., Kamdar N. S., Seiler K., Morgan D. M., Lin P., As-Sanie S. (2018). Definition development and prevalence of new persistent opioid use following hysterectomy. *American Journal of Obstetrics and Gynecology*.

[34] Kuo Y.-F., Raji M. A., Chen N.-W., Hasan H., Goodwin J. S. (2016). Trends in opioid prescriptions among part D medicare recipients from 2007 to 2012. *American Journal of Medicine*.

[35] Clarke H., Soneji N., Ko D. T., Yun L. S., Wijeysundera D. N. (2014). Rates and risk factors for prolonged opioid use after major surgery: population based cohort study. *British Medical Journal*.

[36] Smith A. H., Jensen K. P., Li J. (2017). Genome-wide association study of therapeutic opioid dosing identifies a novel locus upstream of OPRM1. *Molecular Psychiatry*.

[37] De Gregori M., Diatchenko L., Ingelmo P. M. (2016). Human genetic variability contributes to postoperative morphine consumption. *Journal of Pain*.

[38] Doehring A., Küsener N., Flühr K., Neddermeyer T. J., Schneider G., Lotsch J. (2011). Effect sizes in experimental pain produced by gender, genetic variants and sensitization procedures. *PLoS One*.

[39] Levran O., Londono D., O’Hara K. (2008). Genetic susceptibility to heroin addiction: a candidate gene association study. *Genes, Brain and Behavior*.

[40] Kishi T., Yoshimura R., Okochi T. (2010). Association analysis of SIGMAR1 with major depressive disorder and SSRI response. *Neuropharmacology*.

[41] Clarke T.-K., Crist R. C., Kampman K. M. (2013). Low frequency genetic variants in the *μ*-opioid receptor (OPRM1) affect risk for addiction to heroin and cocaine. *Neuroscience Letters*.

[42] Shabalina S. A., Zaykin D. V., Gris P. (2009). Expansion of the human *μ*-opioid receptor gene architecture: novel functional variants. *Human Molecular Genetics*.

[43] Bond C., LaForge K. S., Tian M. (1998). Single-nucleotide polymorphism in the human mu opioid receptor gene alters *β*-endorphin binding and activity: possible implications for opiate addiction. *Proceedings of the National Academy of Sciences*.

[44] Wang S.-C., Tsou H.-H., Chen C.-H. (2012). Genetic polymorphisms in the opioid receptor mu1 gene are associated with changes in libido and insomnia in methadone maintenance patients. *European Neuropsychopharmacology*.

[45] Lettre G., Palmer C. D., Young T. (2011). Genome-wide association study of coronary heart disease and its risk factors in 8,090 African Americans: the NHLBI CARe project. *PLoS Genetics*.

[46] Kringel D., Ultsch A., Zimmermann M. (2017). Emergent biomarker derived from next-generation sequencing to identify pain patients requiring uncommonly high opioid doses. *Pharmacogenomics Journal*.

[47] Donaldson R., Sun Y., Liang D.-Y. (2016). The multiple PDZ domain protein Mpdz/MUPP1 regulates opioid tolerance and opioid-induced hyperalgesia. *BMC Genomics*.

[48] Aubrun F., Zahr N., Langeron O. (2018). Opioid-related genetic polymorphisms do not influence postoperative opioid requirement: a prospective observational study. *European Journal of Anaesthesiology*.

[49] Sun E. C., Darnall B. D., Baker L. C., Mackey S. (2016). Incidence of and risk factors for chronic opioid use among opioid-naive patients in the postoperative period. *JAMA Internal Medicine*.

[50] Khor S., Lavallee D., Cizik A. M. (2018). Development and validation of a prediction model for pain and functional outcomes after lumbar spine surgery. *JAMA Surgery*.

[51] Ditre J., Gonzalez B., Simmons V., Faul L., Brandon T., Jacobsen P. (2011). Associations between pain and current smoking status among cancer patients. *Pain*.

[52] Logan H. L., Fillingim R. B., Bartoshuk L. M. (2010). Smoking status and pain level among head and neck cancer patients. *Journal of Pain*.

[53] Bateman B. T., Franklin J. M., Bykov K. (2016). Persistent opioid use following Cesarean delivery: patterns and predictors among opioid naïve women. *American Journal of Obstetrics and Gynecology*.

[54] Brummett C. M., Waljee J. F., Goesling J. (2017). New persistent opioid use after minor and major surgical procedures in US adults. *JAMA Surgery*.

[55] Aydogan M. S., Ozturk E., Erdogan M. A. (2013). The effects of secondhand smoke on postoperative pain and fentanyl consumption. *Journal of Anesthesia*.

[56] van Erp S. J., Brakenhoff L. K., van Gaalen F. A. (2016). Classifying back pain and peripheral joint complaints in inflammatory bowel disease patients: a prospective longitudinal follow-up study. *Journal of Crohn’s and Colitis*.

[57] Davies-Tuck M. L., Wluka A. E., Forbes A. (2009). Smoking is associated with increased cartilage loss and persistence of bone marrow lesions over 2 years in community-based individuals. *Rheumatology*.

[58] Lahiri M., Morgan C., Symmons D. P. M., Bruce I. N. (2012). Modifiable risk factors for RA: prevention, better than cure?. *Rheumatology*.

[59] Saevarsdottir S., Wedrén S., Seddighzadeh M. (2011). Patients with early rheumatoid arthritis who smoke are less likely to respond to treatment with methotrexate and tumor necrosis factor inhibitors: observations from the epidemiological investigation of rheumatoid arthritis and the swedish rheumatology reg. *Arthritis & Rheumatism*.

[60] Ditre J. W., Heckman B. W., Zale E. L., Kosiba J. D., Maisto S. A. (2016). Acute analgesic effects of nicotine and tobacco in humans: a meta-analysis. *Pain*.

[61] Rydell E., Forslind K., Nilsson J-A., Jacobsson L. T. H., Turesson C. (2018). Smoking, body mass index, disease activity, and the risk of rapid radiographic progression in patients with early rheumatoid arthritis. *Arthritis Research & Therapy,*.

[62] Delgado-Enciso I., Paz-Michel B., Melnikov V. (2017). Smoking and female sex as key risk factors associated with severe arthralgia in acute and chronic phases of chikungunya virus infection. *Experimental and Therapeutic Medicine*.

[63] Wang H., Wang T., Wang Q., Ding W. (2017). Incidence and risk factors of persistent low back pain following posterior decompression and instrumented fusion for lumbar disk herniation. *Journal of Pain Research*.

[64] Momi S., Fabiane S., Lachance G., Livshits G., Williams F. M. (2015). Neuropathic pain as part of chronic widespread pain. *Pain*.

[65] Yosef A., Allaire C., Williams C. (2016). Multifactorial contributors to the severity of chronic pelvic pain in women. *American Journal of Obstetrics & Gynecology*.

[66] Ackerman I. N., Zomer E., Gilmartin-Thomas J. F.-M., Liew D. (2018). Forecasting the future burden of opioids for osteoarthritis. *Osteoarthritis and Cartilage*.

[67] Olfson M., Wall M. M., Liu S.-M., Blanco C. (2018). Cannabis use and risk of prescription opioid use disorder in the United States. *American Journal of Psychiatry*.

[68] Weiser T. G., Haynes A. B., Molina G. (2016). Size and distribution of the global volume of surgery in 2012. *Bulletin of the World Health Organization*.

[69] Blanco C., Wall M. M., Okuda M., Wang S., Iza M., Olfson M. (2016). Pain as a predictor of opioid use disorder in a nationally representative sample. *American Journal of Psychiatry*.

[70] Chaudhary M. A., Schoenfeld A. J., Harlow A. F. (2017). Incidence and predictors of opioid prescription at discharge after traumatic injury. *JAMA Surgery*.

[71] Wick E. C., Grant M. C., Wu C. L. (2017). Postoperative multimodal analgesia pain management with nonopioid analgesics and techniques. *JAMA Surgery*.

[72] Chiang H.-L., Chia Y.-Y., Lin H.-S., Chen C.-H. (2016). The implications of tobacco smoking on acute postoperative pain: a prospective observational study. *Pain Research & Management*.

[73] Lee J. S.-J., Hu H. M., Edelman A. L. (2017). New persistent opioid use among patients with cancer after curative-intent surgery. *Journal of Clinical Oncology*.

[74] Sullivan M. D., Bauer A. M., Fulton-Kehoe D. (2016). Trends in opioid dosing among Washington state medicaid patients before and after opioid dosing guideline implementation. *Journal of Pain*.

[75] de la Cruz M., Reddy A., Balankari V. (2017). The impact of an educational program on patient practices for safe use, storage, and disposal of opioids at a comprehensive cancer center. *The Oncologist*.

[76] Hooten W. M. (2016). Chronic pain and mental health disorders. *Mayo Clinic Proceedings*.

[77] Soneji N., Clarke H. A., Ko D. T., Wijeysundera D. N. (2016). Risks of developing persistent opioid use after major surgery. *JAMA Surgery*.

[78] Andersen K. G., Duriaud H. M., Kehlet H., Aasvang E. K. (2017). The relationship between sensory loss and persistent pain 1 year after breast cancer surgery. *Journal of Pain*.

[79] Sabourdin N., Barrois J., Louvet N. (2017). Pupillometry-guided intraoperative remifentanil administration versus standard practice influences opioid use: a randomized study. *Anesthesiology*.

[80] Edry R., Recea V., Dikust Y., Sessler D. (2016). Preliminary intraoperative validation of the nociception level index: a noninvasive nociception monitor. *Anesthesiology*.

[81] Murata-Ooiwa M., Tsukada S., Wakui M. (2017). Intravenous acetaminophen in multimodal pain management for patients undergoing total knee arthroplasty: a randomized, double-blind, placebo-controlled trial. *Journal of Arthroplasty*.

[82] Haumann J., Geurts J. W., van Kuijk S. M. J., Kremer B., Joosten E. A., van den Beuken-van Everdingen M. H. J. (2016). Methadone is superior to fentanyl in treating neuropathic pain in patients with head-and-neck cancer. *European Journal of Cancer*.

[83] Cheung C. W., Qiu Q., Ying A. C. L., Choi S. W., Law W. L., Irwin M. G. (2014). The effects of intra-operative dexmedetomidine on postoperative pain, side-effects and recovery in colorectal surgery. *Anaesthesia*.

[84] Ouchi K., Sugiyama K. (2016). Dexmedetomidine dose dependently enhances the local anesthetic action of lidocaine in inferior alveolar nerve block: a randomized double-blind study. *Regional Anesthesia and Pain Medicine*.

[85] Nader A., Kendall M. C., Manning D. W. (2016). Single-dose adductor canal block with local infiltrative analgesia compared with local infiltrate analgesia after total knee arthroplasty. *Regional Anesthesia and Pain Medicine*.

[86] Hall-Burton D. M., Hudson M. E., Grudziak J. S., Cunningham S., Boretsky K., Boretsky K. R. (2016). Regional anesthesia is cost-effective in preventing unanticipated hospital admission in pediatric patients having anterior cruciate ligament reconstruction. *Regional Anesthesia and Pain Medicine*.

[87] Derry S., Cooper T. E., Phillips T. (2016). Single fixed-dose oral dexketoprofen plus tramadol for acute postoperative pain in adults. *Cochrane Database of Systematic Reviews*.

[88] Clarke H. A., Katz J., McCartney C. J. L. (2014). Perioperative gabapentin reduces 24 h opioid consumption and improves in-hospital rehabilitation but not post-discharge outcomes after total knee arthroplasty with peripheral nerve block. *British Journal of Anaesthesia*.

[89] Shimony N., Amit U., Minz B. (2016). Perioperative pregabalin for reducing pain, analgesic consumption, and anxiety and enhancing sleep quality in elective neurosurgical patients: a prospective, randomized, double-blind, and controlled clinical study. *Journal of Neurosurgery*.

[90] Dietze P., Cantwell K. (2016). Intranasal naloxone soon to become part of evolving clinical practice around opioid overdose prevention. *Addiction*.

[91] Smith C. A., Levett K. M., Collins C. T., Armour M., Dahlen H. G., Suganuma M. (2018). Relaxation techniques for pain management in labour. *Cochrane Database of Systematic Reviews*.

[92] Sun E. C., Dixit A., Humphreys K., Darnall B. D., Baker L. C., Mackey S. (2017). Association between concurrent use of prescription opioids and benzodiazepines and overdose: retrospective analysis. *British Medical Journal*.

[93] Quinn P. D., Hur K., Chang Z. (2017). Incident and long-term opioid therapy among patients with psychiatric conditions and medications: a national study of commercial health care claims. *Pain*.

[94] Halbert B., Davis R., Wee C. (2016). Disproportionate longer-term opioid use among U.S. adults with mood disorders. *Pain*.

[95] Finkelstein Y., Macdonald E. M., Gonzalez A. (2017). Overdose risk in young children of women prescribed opioids. *Pediatrics*.

[96] Kumar K., Gulotta L. V., Dines J. S. (2017). Unused opioid pills after outpatient shoulder surgeries given current perioperative prescribing habits. *American Journal of Sports Medicine*.

[97] Maughan B. C., Hersh E. V., Shofer F. S. (2016). Unused opioid analgesics and drug disposal following outpatient dental surgery: a randomized controlled trial. *Drug and Alcohol Dependence*.

[98] Dyer O. (2016). Opioid manufacturer bribed doctors to prescribe fentanyl inappropriately, US says. *British Medical Journal*.

[99] Dilokthornsakul P., Moore G., Campbell J. D. (2016). Risk factors of prescription opioid overdose among Colorado medicaid beneficiaries. *Journal of Pain*.

[100] Baker J. A., Avorn J., Levin R., Bateman B. T. (2016). Opioid prescribing after surgical extraction of teeth in medicaid patients, 2000–2010. *JAMA*.

[101] Moyo P., Simoni-Wastila L., Griffin B. A. (2017). Impact of prescription drug monitoring programs (PDMPs) on opioid utilization among medicare beneficiaries in 10 US States. *Addiction*.

[102] Meara E., Horwitz J. R., Powell W. (2016). State legal restrictions and prescription-opioid use among disabled adults. *New England Journal of Medicine*.

[103] Alam A., Gomes T., Zheng H., Mamdani M. M., Juurlink D. N., Bell C. M. (2012). Long-term analgesic use after low-risk surgery. *Archives of Internal Medicine*.

[104] Raebel M. A., Newcomer S. R., Reifler L. M. (2013). Chronic use of opioid medications before and after bariatric surgery. *JAMA*.

[105] Meyer L., Lasala J., Iniesta M. (2018). Effect of an enhanced recovery after surgery program on opioid use and patient-reported outcomes. *Obstetrics & Gynecology*.

[106] Kwan I., Wang R., Pearce E., Bhattacharya S. (2018). Pain relief for women undergoing oocyte retrieval for assisted reproduction. *Cochrane Database of Systematic Reviews*.

[107] Ladha K., Patorno E., Liu J., Bateman B. (2016). Impact of perioperative epidural placement on postdischarge opioid use in patients undergoing abdominal surgery. *Anesthesiology*.

[108] Memtsoudis S., Poeran J., Zubizarreta N. (2018). Association of multimodal pain management strategies with perioperative outcomes and resource utilization. *Anesthesiology*.

[109] Monks D., Hoppe D., Downey K., Shah V., Bernstein P., Carvalho J. C. (2015). A perioperative course of gabapentin does not produce a clinically meaningful improvement in analgesia after cesarean delivery: a randomized controlled trial. *Anesthesiology*.

[110] YaDeau J. T., Lin Y., Mayman D. J. (2015). Pregabalin and pain after total knee arthroplasty: a double-blind, randomized, placebo-controlled, multidose trial. *British Journal of Anaesthesia*.

[111] Barkin R. L., Beckerman M., Blum S. L., Clark F. M., Koh E.-K., Wu D. S. (2010). Should nonsteroidal anti-inflammatory drugs (NSAIDs) be prescribed to the older adult?. *Drugs & Aging*.

[112] Dualé C., Ouchchane L., Schoeffler P. (2014). Neuropathic aspects of persistent postsurgical pain: a French multicenter survey with a 6-month prospective follow-up. *Journal of Pain*.

[113] Jay G. W., Barkin R. L. (2018). Perspectives on the opioid crisis from pain medicine clinicians. *Disease-a-Month*.

[114] Gomes T., Juurlink D. N., Antoniou T., Mamdani M. M., Paterson J. M., van den Brink W. (2017). Gabapentin, opioids, and the risk of opioid-related death: a population-based nested case-control study. *PLoS Medicine*.

[115] Hah J., Mackey S. C., Schmidt P. (2018). Effect of perioperative gabapentin on postoperative pain resolution and opioid cessation in a mixed surgical cohort. *JAMA Surgery*.

[116] Edwards K. A., Havelin J. J., Mcintosh M. I. (2018). A kappa opioid receptor agonist blocks bone cancer pain without altering bone loss, tumor size, or cancer cell proliferation in a mouse model of cancer-induced bone pain. *Journal of Pain*.

[117] Molero Y., Zetterqvist J., Binswanger I. A., Hellner C., Larsson H., Fazel S. (2018). Medications for alcohol and opioid use disorders and risk of suicidal behavior, accidental overdoses, and crime. *American Journal of Psychiatry*.

[118] Nicholson A. B., Watson G. R., Derry S., Wiffen P. J. (2017). Methadone for cancer pain. *Cochrane Database of Systematic Reviews*.

[119] Boland J. W., Pockley A. G. (2018). Influence of opioids on immune function in patients with cancer pain: from bench to bedside. *British Journal of Pharmacology*.

[120] Jones C. M., Baldwin G. T., Manocchio T., White J. O., Mack K. A. (2016). Trends in methadone distribution for pain treatment, methadone diversion, and overdose deaths—United States, 2002–2014. *MMWR. Morbidity and Mortality Weekly Report*.

[121] Hoffman E. M., Watson J. C., St Sauver J., Staff N. P., Klein C. J. (2017). Association of long-term opioid therapy with functional status, adverse outcomes, and mortality among patients with polyneuropathy. *JAMA Neurology*.

[122] Duale C. (2014). Prolonged use of opioids after surgery. *British Medical Journal*.

[123] Olfson M., Crystal S., Wall M., Wang S., Liu S.-M., Blanco C. (2018). Causes of death after nonfatal opioid overdose. *JAMA Psychiatry*.

[124] Abid Azam M., Aliza Z. W., Montbriand J. (2017). Acceptance and commitment therapy to manage pain and opioid use after major surgery: preliminary outcomes from the toronto general hospital transitional pain service. *Canadian Journal of Pain*.

[125] Weinrib A. Z., Azam M. A., Birnie K. A., Burns L. C., Clarke H., Katz J. (2017). The psychology of chronic post-surgical pain: new frontiers in risk factor identification, prevention and management. *British Journal of Pain*.

[126] Chou R., Korthuis P. T., McCarty D. (2017). Management of suspected opioid overdose with naloxone in out-of-hospital settings. *Annals of Internal Medicine*.

[127] Kolodny A., Frieden T. R. (2017). Ten steps the federal government should take now to reverse the opioid addiction epidemic. *JAMA*.

[128] Hurd Y. L., O’Brien C. P. (2018). Molecular genetics and new medication strategies for opioid addiction. *American Journal of Psychiatry*.

[129] Olson M. E., Janda K. D. (2018). Vaccines to combat the opioid crisis. *EMBO Reports*.

